# Long-Term Efficacy of Insecticidal Wall Painting for Controlling Visceral Leishmaniasis Vectors in Bangladesh

**DOI:** 10.4269/ajtmh.22-0809

**Published:** 2023-09-18

**Authors:** Abdul Alim, M Mamun Huda, Debashis Ghosh, Christine M. Halleux, Md. Almahmud, Piero L. Olliaro, Greg Matlashewski, Axel Kroeger, Abraham Aseffa, Dinesh Mondal

**Affiliations:** ^1^ Nutrition and Clinical Services Division, International Centre for Diarrhoeal Diseases Research, Bangladesh, Dhaka, Bangladesh;; ^2^ Poche Centre for Indigenous Health, The University of Queensland, Brisbane, Australia;; ^3^ ARC Centre of Excellence for Children and Families over the Life Course, The University of Queensland, Brisbane, Australia;; ^4^ UNICEF/UNDP/World Bank/World Health Organization Special Programme for Research and Training in Tropical Diseases (TDR), World Health Organization, Geneva, Switzerland;; ^5^ ISARIC Global Support Centre, International Severe Acute Respiratory and Emerging Infection Consortium, Pandemic Sciences Institute, Nuffield Department of Medicine, University of Oxford, Oxford, United Kingdom;; ^6^ Department of Microbiology and Immunology, McGill University, Montreal, Canada;; ^7^ Centre for Medicine and Society/Institute for Infection Prevention, University Medical Centre, Freiburg, Germany

## Abstract

The success of the visceral leishmaniasis (VL) elimination program largely depends on cost-effective vector control measures. Our goal was to investigate the longevity of the efficacy of insecticidal wall painting (IWP), a new vector control tool, compared with a routine indoor residual spraying (IRS) program for reducing the VL vector density in Bangladesh. This study is the extension of our recent IWP study for VL vector management in Bangladesh, which was undertaken in seven highly VL endemic villages of the Mymensingh district with a 12-month follow-up. In this 24-months follow-up study, we collected sand flies additionally at 15, 18, 21, and 24 months since the interventions from the IWP and control (where the program did routine IRS) clusters to examine the longevity of the efficacy of IWP on sand fly density reduction and mortality. The difference-in-differences regression models were used to estimate the effect of IWP on sand fly reduction against Program IRS. The IWP showed excellent performance in reducing sand fly density and increasing sand fly mortality compared with Program IRS. The effect of IWP for controlling sand flies was statistically significant for up to at least 24 months. The mean female *Phlebotomus argentipes* density reduction ranged from −56% to −83%, and the *P. argentipes* sand fly mortality ranged from 81% to 99.5% during the 24-month follow-up period. Considering the duration of the efficacy of IWP for controlling VL vectors, Bangladesh National Kala-azar Elimination Program may consider IWP as the best alternative to IRS for the subsequent phases of the program.

## INTRODUCTION

Visceral leishmaniasis (VL, Kala-azar) is a crucial problem for public health worldwide because it is potentially lethal if untreated. The parasite *Leishmania donovani* causes VL in Bangladesh and the female *Phlebotomus argentipes* is its vector which transmits the parasites among community people. First reported VL outbreak in the Bangladesh territory occurred in 1824 when ∼75,000 people died.^1^ In 2005, the governments of India, Bangladesh, and Nepal committed to eliminate VL from the Indian subcontinent by 2015. The elimination target was the number of cases being less than 1 per 10,000 people at the Public Health Center (PHC) block, upazila, district level in India, Bangladesh, and Nepal, respectively.^2^ Nepal and Bangladesh achieved the elimination target in 2013 and 2016, respectively. However, Nepal could not keep the target for 3 consecutive years. India also made significant progress and was close to achieving the target, with only eight PHC blocks having VL cases > 1 per 10,000 people in 2020.^3^^,^^4^ The elimination program had five main pillars in the attack phase, such as early diagnosis and complete case management, integrated vector management and vector surveillance, effective disease surveillance through passive and active case detection, social mobilization and building partnerships, and implementation and operational research.^5^ The subsequent phases of the program, the consolidation and maintenance phases, require an effective vector control intervention. Due to the current low number of VL cases, cost-effective measures with sustained effects are essential; therefore, the National Kala-azar Elimination Program (NKEP) is interested in alternative vector control measures to indoor residual spraying (IRS), which is operationally challenging and expensive to maintain. Currently available alternatives for IRS as vector control methods includes insecticide-treated bed nets (ITN),^6^^,^^7^ durable wall lining (DWL; 1.5 m or 1.8 m in height),^8^ and treatment of vector larvae through deployment of larvicide at suspected sand fly breeding places around household (HH) in the endemic villages.^5^ In Bangladesh, all these interventions are effective but have different costs and produce effects of variable duration. In Africa, a new tool named insecticidal wall painting (IWP) has been found to be very effective to control mosquito.^9^^,^^10^ Later IWP was also found effective for sand fly control in Nepal.^11^ In Bangladesh, IWP performed better than ITN, IRS, and DWL.^12^ Therefore, IWP could be an effective alternative tool for vector control compared with the existing vector control options. Considering the lack of evidence of the long-term effectiveness of IWP compared with the existing vector control options, there is a need to investigate the longevity of the effects of IWP. Therefore, we conducted this follow-up study to compare the performance of IWP for up to 24 months against the existing IRS in the elimination program in Bangladesh.

## MATERIALS AND METHODS

### Study intervention and comparator.

#### Insecticidal wall painting.

Details of the IWP intervention have been described elsewhere.^12^ Briefly, the trained field research assistant painted all the study HHs with Inesfly 5AIGRNG^TM^ with a coverage of 2.43 m^2^ of the HH wall. Inesfly 5AIGRNG contains alphacypermethrin 0.7%, D-allethrin 1.0%, and pyriproxyfen (0.063%). It is a vinyl paint with an aqueous base formulation with CaCO_3_ and resin microcapsules as active ingredients, which allow a gradual release of active ingredients. The size of the microcapsules ranges from one to several hundred micrometers. After painting, the concentration of the insecticides per square meter surface area of the wall should be 1.225, 1.75, and 0.11 g/m^2^ for alphacypermethrin, D-allethrin, and pyriproxyfen, respectively. There were 56 HHs in this experimental arm. The interventions were imposed in November 2015 and followed up until December 2017.

#### Program IRS.

The routine IRS program in NKEP was this study’s reference arm (comparator). The program’s trained team sprayed up to 6 feet (180 cm) of the indoor walls of the HHs of the study villages with deltamethrin 5 WP (Tagros Chemical India Ltd., Chennai, India) before 6- and 18-month follow-ups. The program IRS arm had 54 HHs and presented as reference arm (comparator) in this study.

#### Study areas and duration.

The Trishal upazila (subdistrict) under Mymensingh district was our study area. The study was carried out from November 2015 to December 2017. Trishal is the highest VL endemic upazila of Bangladesh.^5^ Of the 12 unions of this upazila, there were five VL endemic unions. For this study, we selected Sakhua union (seven villages) based on the past 12 months of hospital case reports.

#### Study design.

Detailed study design has been published elsewhere.^12^ Briefly, Sakhua union had seven villages with HHs ranging from 308 to 1,581. We divided each village into clusters with a size of approximately 50 HHs in each cluster. We randomly selected one cluster from each village. Two to 4 weeks before interventions, baseline sand fly density measurement was done in all seven clusters. Among the seven clusters, we randomly selected four clusters; likewise, the selection of the type of intervention and control cluster (either IWP or control) was random. As per the minimum required number of HHs needed for sand fly density monitoring,^12^ 36 HHs from the intervention cluster and 36 HHs from the control cluster were selected randomly for entomological activities. The NKEP conducted routine IRS in the control village, which acted as a comparator. In this follow-up study, we investigated the duration of the effects of IWP against Program IRS for controlling sand fly density and for sand fly mortality for up to 24 months.

#### Entomological activities and methods.

Trained ento-technicians collected sand flies from the randomly selected 36 HHs from intervention cluster and 36 HHs from Program IRS cluster 2 to 4 weeks before intervention (baseline), and at 1, 3, 6, 9, 12, 15, 18, 21, and 24 months since the intervention. We assessed sand fly mortality through the WHO cone bioassay test in 12 HHs in the intervention cluster at 1, 3, 6, 9, 12, 15, 18, 21, and 24 months since the intervention. Collection of sand fly and density measurement and the WHO cone bioassay test were performed according to the methods described in our recent study.^12^

#### Efficacy measurement of IWP.

We used two methods to calculate the effect of IWP on sand fly control at HH level using: 1) sand fly density reduction (in percentage) by IWP compared with Program IRS and 2) sand fly mortality rate (in percentage) when exposed to IWP compared with the control.

### Statistical analysis.

Data entry was conducted using the Epi Info software (Version 3.5) for data entry and data management. Before data analysis, data were cleaned with checks for duplication and inconsistency. We dichotomized sociodemographic and economic variables. For calculation of HH asset score, we used principal component analysis. Descriptive analysis was performed. Sociodemographic and economic variables were compared between the two groups using the χ^2^ test. The mean HH female *P. argentipes* sand fly values in the cluster defined the female *P. argentipes* sand fly density (FPAD) of a cluster.

The IWP effect on sand fly density at the HH level against Program IRS was measured by the difference-in-difference (DID) regression model. It accounted for the baseline differences of FPAD and for the variations between IWP and Program IRS regarding their covariates. An interaction term for the intervention cluster at follow-up estimated the intervention effect. The regression follows:

Number of FPA=Intercept+a*Treatment+b*Time+c*Interactions+error,

where treatment is 1 if it is the IWP and 0 if it is the Program IRS; where time is 1 if follow-up and 0 if baseline; and where interaction is 1 for the IWP group at follow-up.

First, we fitted the unadjusted regression model by considering only the time and treatment (IWP versus Program IRS) variables in the model to get the unadjusted intervention effect. The model was then extended further by incorporating the variables that differed significantly between IWP and Program IRS clusters to get the adjusted intervention effect. On the basis of the bivariate analysis (Table 1), in the adjusted model, we included the following variables: head of HH occupation, head of HH education, number of bedrooms, ownership of cattle shed, cow in the HH, goat in the HH, chicken in the HH, and mosquito coil and bed net use. We measured the intervention effect using DID in the average FPAD generated from the c-coefficient in the model. When c-coefficient was negative, it indicated that the sand fly density had decreased; therefore, the IWP was more effective, and vice versa. The zero c-coefficient demonstrated that there was no difference in changes of the sand fly density between IWP and Program IRS. Percent reduction of FPAD by IWP relative to Program IRS is calculated and given with 95% CI:

Percent reduction of FPAD by IWP=(c−coefficient/baseline FPAD of IWP)*100

**Table 1:** Comparison of HH sociodemographic characteristics between IWP and Program IRS arms at baseline

Variables	IWP	Program IRS	*P* value
	% (*n*), *N* = 36	% (*n*), *N* = 36	
Labor HHH	30.56 (11)	16.67 (6)	0.165
Illiterate HHH	44.44 (16)	61.11 (22)	0.157
Number of bedrooms < 2	30.56 (11)	16.67 (6)	0.165
Having veranda in the HH	19.44 (7)	22.22 (8)	0.772
Having Cattle shed in the HH	30.56 (11)	69.44 (25)	0.001
Low asset score	27.78 (10)	16.67 (6)	0.257
Having cow in the HH	30.56 (11)	72.22 (26)	<0.0001
Having goat in the HH	22.22 (8)	38.89 (14)	0.125
Having chicken in the HH	77.78 (28)	94.44 (34)	0.085
Having duck in the HH	47.22 (17)	58.33 (21)	0.345
No. of bed net < 2	41.67 (15)	27.78 (10)	0.216
Use of mosquito coil	13.89 (5)	30.56 (11)	0.089
Mud wall	19.44 (7)	22.22 (8)	0.772
Crack wall	19.44 (7)	22.22 (8)	0.772
Mud floor	83.33 (30)	88.89 (32)	0.735
Always use bed net	58.33 (21)	19.44 (7)	0.001
Baseline FPAD mean (95% CI)	1.64 (1.01 to 2.27)	0.56 (0.17 to 0.94)	0.004

FPAD = female *P. argentipes* sand fly density; HH = household; HHH = household head; IRS = indoor residual spraying; IWP = insecticidal wall painting.

We considered an intervention effective for killing sand flies if Abbot’s corrected sand fly mortality remained ≥ 80%.

## RESULTS

At baseline, the mean [95% CI] FPAD at HH level was significantly higher in IWP cluster (1.64 [1.01–2.27]) than Program IRS cluster (0.56 [0.17–0.94]) (*P* = 0.004) (Table 1). We also found that three of the HH characteristics such as ownership of a cattle shed (*P* = 0.001), ownership of cow in the HH (*P* < 0.0001), and bed-net use (*P* = 0.001) significantly differed between IWP and Program IRS at baseline (Table 1). For example, in 58.3% of HHs in the IWP cluster, HH members often used bed nets to protect themselves from sand flies or mosquitoes, whereas this was only 19.44% in the Program IRS cluster (*P* = 0.001) (Table 1).

**Figure 1.**
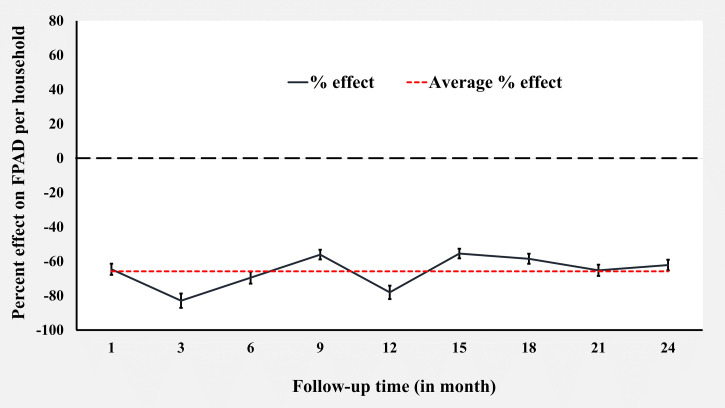
Effect of insecticidal wall painting on female *Phlebotomus argentipes* sand-fly density (FPAD) per household against Program indoor residual spraying.

Comparing FPAD between baseline and follow-up, we found that the reduction of mean FPAD was higher in the IWP cluster than in Program IRS cluster at 24 months of follow-up. In the IWP cluster, the mean FPAD dropped from 1.64 to 0.19 per HH at 24-month follow-up, whereas for Program IRS, it dropped from 0.56 to 0.14 per HH (Table 2, Figure 1). The unadjusted DID regression model showed that IWP significantly reduced the mean FPAD at the HH level up to 24 months compared with Program IRS (percent reduction −62.80%, 95% CI: −175.25 to −12.78). When adjusted for the potential confounding covariates with *P* < 0.20 in bivariate analysis (Table 1) such as HH head (HHH) occupation; HHH education; number of bedrooms; ownership of cattle shed; cow, goat, or chicken in the HH; and the use of mosquito coil and bed net use in the HH did not alter the effect of IWP. The effect remained statistically significant, with an adjusted percent reduction of mean FPAD of −62.20% (95% CI: −175.25 to −11.89) compared with Program IRS (Table 2). During the whole study period, the average FPAD reduction was −65.85% (95% CI: −142.57% to −31.72%), varying from −82.93% (95% CI: −214.85 to −24.23) to −55.49% (95% CI: −170.30 to −4.41) (Table 2, Figure 1).

**Table 2:** Female *P. argentipes* sand fly per HH and their comparison between IWP and Program IRS arms at baseline and follow-up

Time	Female *P. argentipes* sand fly per household, mean (95% CI)		Effect in % (95% CI) on FPAD by IWP against Program IRS	
	IWP (*N* = 36)	Program IRS (*N* = 36)	Unadjusted	Adjusted*
Baseline	1.64 (1.01 to 2.27)	0.56 (0.17 to 0.94)	–	–
1-month follow-up	0.03 (−0.03 to 0.08)	0.00 (0.00 to 0.00)	−64.63 (−176.24 to −14.54)	−64.63 (−177.23 to −14.10)
3-month follow-up	0.31 (0.11 to 0.50)	0.58 (0.26 to 0.91)	−82.93 (−214.85 to −24.23)	−82.93 (−214.85 to −24.23)
6-month follow-up	0.31 (0.13 to 0.48)	0.36 (0.13 to 0.59)	−69.51 (−189.11 to −15.86)	−69.51 (−191.09 to −15.42)
9-month follow-up	0.36 (0.12 to 0.61)	0.19 (−0.02 to 0.41)	−56.10 (−168.32 to −5.73)	−56.10 (−169.31 to −5.29)
12-month follow-up	0.19 (0.06 to 0.33)	0.39 (0.11 to 0.67)	−78.05 (−203.96 to −22.03)	−78.05 (−204.95 to −21.15)
15-month follow-up	0.36 (0.07 to 0.65)	0.19 (0.02 to 0.37)	−56.10 (−169.31 to −5.29)	−55.49 (−170.30 to −4.41)
18-month follow-up	0.22 (0.04 to 0.41)	0.11 (0.00 to 0.22)	−59.15 (−170.30 to −9.69)	−58.54 (−170.30 to −9.25)
21-month follow-up	0.14 (0.02 to 0.26)	0.14 (−0.005 to 0.28)	−65.85 (−181.19 to −14.98)	−65.24 (−181.19 to −14.10)
24-month follow-up	0.19 (0.06 to 0.33)	0.14 (0.02 to 0.26)	−62.80 (−175.25 to −12.78)	−62.20 (−175.25 to −11.89)
Average (1–24 month follow-up)	0.23 (0.17 to 0.29)	0.23 (0.17 to 0.30)	−65.85 (−142.57 to −32.16)	−65.85 (−142.57 to −31.72)

FPAD = female *P.argentipes* sand fly density; HH = household; IRS = indoor residual spraying; IWP = insecticidal wall painting.

* Adjusted covariates: household head occupation, household head education, number of bedrooms, having cattle shed, having cow in the household, having goat in the household, having chicken in the household, use of mosquito coil, and use of bed net.

**Figure 2.**
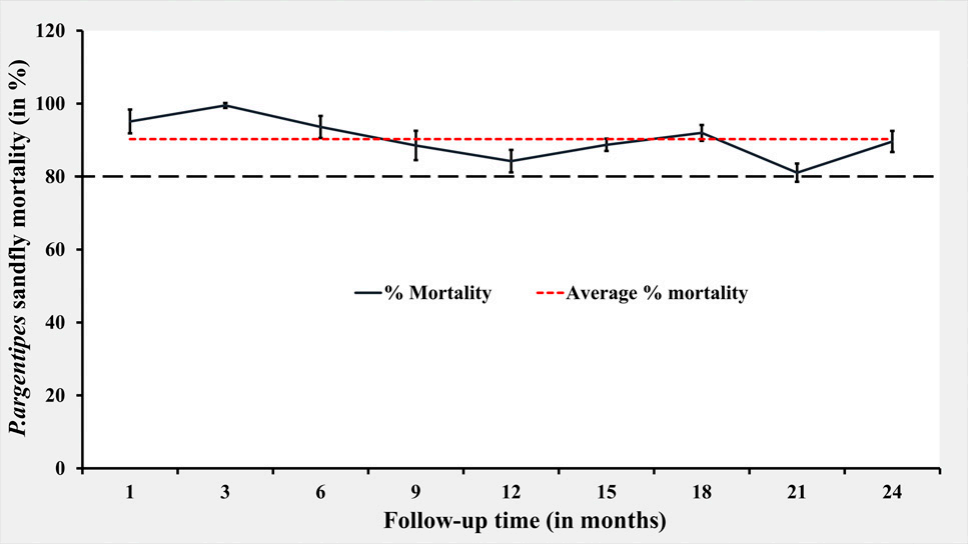
Abbot corrected *Phlebotomus argentipes* sand fly mortality by insecticidal wall painting.

IWP also showed excellent performance regarding sand fly mortality during the 24-month follow-up period ranged from 81% to 99.5%, thus above the minimum level of 80% mortality^12^ (Table 3, Figure 2). We directly observed up to 24 months since intervention and found that 98% of the HHs of the IWP arm were physically intact.

**Table 3:** Abbot-corrected *P. argentipes* sand fly mortality by IWP and follow-up

Time	Average corrected *P. argentipes* sand fly mortality (95% CI) by IWP
1-month follow-up	95.12% (91.54–98.70%)
3-month follow-up	99.50% (98.74–100%)
6-month follow-up	93.60% (90.25–96.94%)
9-month follow-up	88.52% (84.09–92.94%)
12-month follow-up	84.24% (80.86–87.62%)
15-month follow-up	88.71% (86.85–90.56%)
18-month follow-up	91.98% (89.61–94.35%)
21-month follow-up	81.06% (78.31–83.81%)
24-month follow-up	89.61% (86.42–92.79%)
Average (1–24 month follow-up)	90.26% (88.92–91.59%)

IWP = insecticidal wall painting.

## DISCUSSION

The most important finding of the current study is that the IWP continues to be efficacious at 24 months after intervention for controlling sand fly. This study extends the findings of our previous study, in which we compared the efficacy of IWP with all other available vector control tools such as DWL, ITN, and controlled IRS (done by the research team).^12^ In that study, we reported our 12-month observation after interventions and found IWP effective and superior to DWL, ITN, and IRS to controlling sand fly. IWP was also safe, cost-effective, and well accepted by the community.^12^ A study from Nepal also found that IWP was effective for sand fly control for up to 12 months or longer.^11^ This study aimed to examine the longevity of the efficacy of IWP, a new vector control tool, compared with the existing Program IRS for reducing HHs’ VL vector density in VL-endemic areas in Bangladesh. We found that IWP is more effective in terms of reduction of sand fly density in the community as well as sand fly killing effect in the bioassay cone test up to at least 24 months since the intervention compared with the existing IRS of the NKEP in Bangladesh. Investigating efficacy of IWP against Program IRS makes the comparison more meaningful because it reflects efficacy of IWP against IRS in real-life settings and paves the way for IWP for policy translation. The 24-month efficacy of IWP makes it very attractive for sand fly control for the subsequent phases of the NKEP.

In Bangladesh, the NKEP has reached the elimination target through coordinated interventions and considerable investment, with only 35 VL cases in 2021. Sustainable interventions that are adapted to the low case load must now be set in place in the maintenance phase, or these achievements might be lost, and we will face a resurgence of VL.

IRS is resource-intensive, and its efficacy last only 4 to 6 months.^12^^,^^13^ Community people cannot conduct IRS independently, and it needs a coverage of at least 80% in a given area to be effective. IWP use can overcome those operational limitations of the IRS with better efficacy and longevity of efficacy.

Regarding the efficacy of other vector control tools such as ITNs, one study found that ITNs are effective for up to 18 months.^14^ However, in our recent study, the efficacy of ITNs was only up to 6 months.^12^ Another recent study in Nepal demonstrated that ITNs are effective for only 1 month.^11^ According to the literature, one explanation for this variation in longevity of the efficacy of ITN could be the scale of intervention. The long-term efficacy of ITN is observed for mass-scale ITN implementation,^14^ whereas duration is reduced for small-scale implementation (i.e., sporadic or cluster based). Therefore, mass-scale intervention might be crucial for ITN to achieve the expected benefit. Therefore, alternative vector control tools are needed, particularly in the maintenance phase of the NKEP, when mass-scale interventions that are safe, affordable, cost-effective, and sustainable methods for controlling the VL vector are not realistic, and IWP is promising considering all these criteria.

DWL is another effective VL vector control tool in the Indian subcontinent.^5^^,^^8^^,^^15^ However, its long-term efficacy has yet to be explored. DWL is challenging to handle and is expensive for the community and the control program.

The current price of IWP (30 USD per HH) and DWL (50 USD per HH) for full coverage is comparatively high for communities and national programs. The partial coverage with DWL makes its cost closer to that of IWP but the duration of the efficacy of IWP for controlling VL vector is up to 24 months. Furthermore, DWL is no longer in production.

On the other hand, IWP is easy to operate, and intervention can be implemented in the community by the community members themselves. Our recently completed study for IWP feasibility and effectiveness for sand fly control when it is deployed by community members under the supervision of public health workers found encouraging results (unpublished data). Therefore, our present study and the existing body of evidence in the literature clearly indicate that IWP outperforms the existing vector control tools in terms of its feasibility, affordability, and long-term effectiveness.

Our study is not without its limitations. First, as reported earlier,^12^ NKEP deployed the routine IRS cycle during the follow-up phase of this study, so we could not include an untreated control cluster. Thus, we compared the IWP clusters with the control cluster where routine IRS was done during the study (Program IRS). Therefore, future study is required to understand the long-term efficacy of IWP compared with a fresh control. However, because the IWP was found effective compared with the Program IRS up to 24 months, it should also be effective against a fresh control. Second, we should be cautious in interpreting the efficacy of IWP against Program IRS because there were only two IRS during the 24-month follow-up study, and this means two cycles of IRS per annum was not done in the Program IRS arm of this study. Also, the quality of the IRS was not assessed. Third, this study was done in a highly VL endemic small community (a cluster of 56 HHs), where sand fly density is usually high. Therefore, the IWP effect size may not be generalizable to other communities where the vector density is low. Finally, the low VL burden in Bangladesh did not allow us to investigate the effect of the interventions on VL case reduction.

In conclusion, all existing vector control tools, including IWP, are effective, but duration of efficacy is different. In this study on the long-term efficacy of IWP, we found that IWP is effective up to at least 24 months for sand fly density reduction and sand fly mortality. We recommend that the NKEP consider IWP as a first choice VL vector control tool against IRS for the subsequent phases of the VL elimination program in Bangladesh.
